# Barriers and facilitators for interventions to improve ART adherence in Sub-Saharan African countries: A systematic review and meta-analysis

**DOI:** 10.1371/journal.pone.0295046

**Published:** 2023-11-30

**Authors:** Amos Buh, Raywat Deonandan, James Gomes, Alison Krentel, Olanrewaju Oladimeji, Sanni Yaya

**Affiliations:** 1 Interdisciplinary School of Health Sciences, University of Ottawa, Ottawa, Ontario, Canada; 2 School of Epidemiology and Public Health, University of Ottawa, Ottawa, Ontario, Canada; 3 Department of Public Health, Faculty of Health Sciences, Walter Sisulu University, Mthatha, Eastern Cape, South Africa; 4 Faculty of Health Sciences, Durban University of Technology, Durban, South Africa; 5 School of International Development and Global Studies, University of Ottawa, Ottawa, Ontario, Canada; Zimbabwe Health Interventions, ZIMBABWE

## Abstract

**Background:**

The HIV/AIDS pandemic remains a significant public health issue, with sub-Saharan Africa (SSA) at its epicentre. Although antiretroviral therapy (ART) has been introduced to decrease new infections and deaths, SSA reports the highest incidence of HIV/AIDS, constituting two-thirds of the global new infections. This review aimed to elucidate the predominant barriers and facilitators influencing ART adherence and to identify effective strategies to enhance ART adherence across SSA.

**Methods:**

A comprehensive review was conducted on studies examining barriers to ART adherence and interventions to boost adherence among HIV-positive adults aged 15 and above in SSA, published from January 2010 onwards. The research utilized databases like Medline Ovid, CINAHL, Embase, and Scopus. Included were experimental and quasi-experimental studies, randomized and non-randomized controlled trials, comparative before and after studies, and observational studies such as cross-sectional, cohort, prospective and retrospective studies. Two independent reviewers screened the articles, extracted pertinent data, and evaluated the studies’ methodological integrity using Joanna Briggs Institute’s standardized appraisal tools. The compiled data underwent both meta-analysis and narrative synthesis.

**Results:**

From an initial pool of 12,538 papers, 45 were selected (30 for narrative synthesis and 15 for meta-analysis). The identified barriers and facilitators to ART adherence were categorized into seven principal factors: patient-related, health system-related, medication-related, stigma, poor mental health, socioeconomic and socio-cultural-related factors. Noteworthy interventions enhancing ART adherence encompassed counselling, incentives, mobile phone short message service (SMS), peer delivered behavioural intervention, community ART delivery intervention, electronic adherence service monitoring device, lay health worker lead group intervention and food assistance. The meta-analysis revealed a statistically significant difference in ART adherence between the intervention and control groups (pooled OR = 1.56, 95%CI:1.35–1.80, p = <0.01), with evidence of low none statistically significant heterogeneity between studies (*I*^*2*^ = 0%, p = 0.49).

**Conclusion:**

ART adherence in SSA is influenced by seven key factors. Multiple interventions, either standalone or combined, have shown effectiveness in enhancing ART adherence. To optimize ART’s impact and mitigate HIV’s prevalence in SSA, stakeholders must consider these barriers, facilitators, and interventions when formulating policies or treatment modalities. For sustained positive ART outcomes, future research should target specific underrepresented groups like HIV-infected children, adolescents, and pregnant women in SSA to further delve into the barriers, facilitators and interventions promoting ART adherence.

## Background

The HIV/AIDS pandemic remains a pervasive global public health challenge. From the epidemic’s inception until the close of 2021, approximately 84.2 million individuals (range: 64.0–113.0 million) contracted HIV, resulting in an estimated 40.1 million deaths (range: 33.6–48.6 million) due to the virus [1]. In 2021 alone, there were 38.4 million people living with HIV, 1.5 million new infections, and between 510,000 and 860,000 AIDS-related deaths [1, 2]. Sub-Saharan Africa (SSA), or the World Health Organization (WHO) African region, continues to bear the heaviest HIV/AIDS burden. Here, nearly 1 in every 25 adults (3.4%) is living with HIV, comprising over two-thirds of the global HIV population [1, 3].

After an HIV diagnosis, timely and effective linkage to care is pivotal [4, 5]. Currently, there is no cure for HIV. Anti-retroviral therapy (ART) remains the sole treatment that can prolong life and improve the quality of life of people living with HIV/AIDS (PLWHA) [5, 6]. ART has revolutionized the management of HIV, transforming a once-fatal disease into a manageable chronic condition [7]. This therapy inhibits the virus’ replication, reduces the patient’s viral load, increases CD4 counts, and thus decreasing the patient’s risk of opportunistic infections and hospitalizations. This boosts patient’s quality of life and reduces mortality [8–10]. As the patient’s CD4 counts rises, their immune system is rejuvenated, effectively combating infections and HIV-related cancers [6]. For ART to be effective, it must be consistently taken as prescribed [5, 7–9]. When properly adhered to, ART minimizes the individual’s viral load, prevents drug resistance, reduces the risk of transmission, and lowers treatment failure rates [6, 10–12]. Conversely, inconsistent adherence can lead to drug resistance and compromised treatment efficacy [6].

Despite ART’s potential benefits—enabling immune recovery and improving survival in PLWH [11], healthcare systems across SSA grapple with multiple challenges in scaling up ART provision. Key issues include suboptimal ART adherence, poor retention of PLWHA in care, and overloaded primary health care facilities [13]. Such challenges might be contributing to observed ART non-adherence, particularly in regions with high disease prevalence [14]. Research has indicated that patient factors, the nature of the disease, treatment modalities, and the patient-healthcare provider relationship can impact adherence [15, 16]. Notable barriers encompass stigma, negative perceptions, lack of family and community support, status disclosure issues, unemployment, transportation challenges, insufficient nutrition, inadequate follow-ups, confidentiality concerns, and dependency on alternative therapies [17, 18]. Also, physical, economic, and emotional stress, travel away from home, business with other things, depression, alcohol or drug use, and ART dosing frequency have all been identified as barriers to adherence [19, 20]. On the positive side, several facilitators to ART adherence have been documented, including social support [21, 22], HIV status disclosure, health improvement due to ART, use of reminder aids and receiving education and counselling [21, 23]. Community knowledge and understanding of the HIV infection, increasing collaboration between Western and Traditional providers, peer and family level support, decreasing cost and distance to ART clinic [24] as well as clear instructions for taking ART, service providers’ positive attitude towards patients, benefits of adhering to ART and dangers of defaulting [23] have also been documented as factors facilitating ART adherence. Despite this plethora of research, comprehensive insights into the predominant barriers and facilitators common across all SSA countries remain sparse.

Patients on ART frequently face multifaceted barriers to consistent adherence, implying that a single intervention strategy may not suffice. Healthcare providers are thus encouraged to adopt a multifaceted approach—identifying at-risk patients and then tailoring support to address specific adherence obstacles [25]. Documented interventions include home-based care, peer support, and specific treatment regimens [26]. Also, cognitive behavioural interventions, education, treatment supporters, directly observed therapy, and active adherence reminder devices (such as mobile phone text messages) can help patients stick to their ART regimen [27]. However, comprehensive data on interventions universally effective across SSA is scanty.

Considering the acute HIV burden in SSA, coupled with often under-resourced healthcare systems, understanding common barriers to and effective interventions for ART adherence is vital. Such insights can aid countries and organizations in framing effective strategies to curtail the pandemic in the region. This review, therefore, sought to pinpoint the prevalent barriers and facilitators influencing ART adherence and to unearth universally effective interventions that could enhance ART adherence across SSA.

## Methods

### Study design

This was a systematic review and meta-analysis of published studies that examined the barriers and facilitators to ART adherence and interventions that improved patients’ adherence to ART in SSA countries. The review is reported following the Preferred Reporting Items for Systematic Reviews and Meta-Analyses Protocols (PRISMA-P) criteria (S1 File) [28]. The review’s protocol was registered with the International Prospective Register of Systematic Reviews (PROSPERO number CRD42021262256) and published in the Plos One journal [5].

### Inclusion criteria

To select appropriate studies for this review, we used the PICO (population, intervention, comparator, and outcome) criteria. This allowed us to find and select the right studies that can address our research questions. Our inclusion criteria therefore comprised:

### Population

This review included studies conducted between 2010 and 2023 on adult HIV-positive patients aged 15 or above in SSA.

### Intervention

All studies that assessed the barriers and or facilitators to ART adherence and or evaluated interventions aimed at improving ART adherence among adults PLWH in SSA were included in this review.

### Comparator

The interventions were either in comparison with other strategies to identify the most effective and/or were in comparison to no strategies/interventions (regular basic management).

### Outcomes

The review included studies that assessed the following outcomes:

Primary outcome: Proportion of patients adhering to treatment following implementation of specific strategies.
Secondary outcomes: Proportion of patients retained in care, prevalence of opportunistic infections and or the worsening/severity of the patient’s stage of HIV infection following specific treatment interventions. Included studies measured viral load and CD4 cell counts as an indication of the treatment adherence and efficacy.

### Types of studies

This review encompassed both experimental and quasi-experimental studies from SSA that evaluated barriers to ART adherence and interventions aimed at enhancing such adherence. Included studies consisted of randomized and non-randomized controlled trials, comparative pre-and-post studies, and various observational studies such as cross-sectional, cohort, prospective and retrospective investigations. The scope of this review was limited to studies conducted between 2010 and 2023 and involved adult participants aged 15 years and older.

### Language

Only studies written in English and or French were included in this systematic review.

### Search strategy

A three-step strategy was used to find published studies on barriers to ART adherence and interventions improving adherence to ART among adult PLWH in SSA. An initial search through the Medline Ovid database was first conducted using an analysis of text words found in the title and abstract, and the index terms used in describing the article. Secondly, keywords and index terms were identified to search for studies in selected databases. Finally, additional studies not found in the databases were looked for using the reference list of selected studies from the first and second searches. For this review, the databases that were searched included Medline Ovid, CINAHL, Embase, and Scopus. We also used search engines and directories such as Google scholar and Centres for Disease Control and Prevention (CDC) to search for unpublished studies.

Some of the keywords we used for our initial searches in Medline Ovid included *“ART”*, *“adherence”*, *“retention in care”*, *“non-adherence”*, *“barriers to ART adherence”*, *“adherence strategies”*, *“HIV”*, *“adults PLWH”*, *“sub-Saharan African countries”* (S2 File).

### Study screening and selection

Studies that were identified in searched databases were saved in Zotero and exported to the Covidence software for screening. The inclusion and exclusion criteria of this study was also imported to the Covidence software, and the software was used for title, abstract and full-text screening. After importing references and inclusion/exclusion criteria into the software, two independent reviewers screened titles of included studies following the eligibility criteria. All conflicts between the two reviewers were resolved either through discussion or by a third reviewer. This same procedure was applied for abstract screening. After the abstract screening, full texts of potentially eligible studies were retrieved and independently assessed for eligibility by two reviewers. Any conflicts or disagreement between the two reviewers over the eligibility of a given study were resolve in a similar manner as for the title and abstract screening.

### Assessment of methodological quality

Two independent reviewers were used to assess the methodological validity of the studies that were selected for retrieval using standard critical appraisal tools from the Joanna Briggs Institute for Meta-Analysis of Statistics Assessment and Review Instrument (JBI-MAStARI) (S3 File). Any disagreement between the two reviewers were settled through discussions or by a third reviewer.

### Data extraction

Data was extracted from selected studies using a standardized data extraction tool from the Joanna Briggs Institute Meta-Analysis of Statistics Assessment and Review instrument (S4 File). The extracted data included specific details about the barriers to ART adherence, strategies or interventions improving ART adherence, study populations, study methods and outcomes significant to the review question and objective. In the event of any missing data from a study, the corresponding author of the study was contacted to provide the missing data. The data were independently extracted by two reviewers.

### Data synthesis

We conducted both a meta-analysis and a narrative synthesis of the various interventions improving ART adherence. The meta-analysis was done to identify the interventions with a significant impact in improving patients’ adherence to ART.

For the meta-analysis, we first assessed the statistical heterogeneity with I^2^, which indicates the percentage of the total variation across studies; where 0% - 40% indicates low heterogeneity, 30% - 60% indicates moderate heterogeneity, 50% - 90% indicates substantial heterogeneity, and 75% - 100% indicates considerable heterogeneity. If there was a substantial amount of heterogeneity (75%), then sources of heterogeneity were examined through subgroup and sensitivity analyses. We also used Chi-square test to test the heterogeneity and considered P-values < 0.05 as statistically significant. We selected a fixed-effects model for significant homogeneous studies; otherwise, we applied a random-effects model. All outcomes were summarized using odds ratios (OR) and 95% confidence intervals (CI). An OR<1 indicated a lower rate of outcome among the group of patients who were treated following a given intervention. Publication bias was assessed by visual inspections of funnel plots and Egger’s test.

For the narrative synthesis, we have described the barriers and facilitators to ART adherence and the interventions promoting adherence to ART, following Popay’s guidance on the conduct of a narrative synthesis [29]. The narrative synthesis has been structured by describing the studies following the barriers and facilitators assessed and the type of strategies used to improve ART adherence. The identified barriers and facilitators to ART adherence and the interventions improving adherence have first been presented in a table followed by detailed description of each ART adherence barrier and or facilitator and intervention promoting treatment adherence.

### Confidence in cumulative evidence

The quality of evidence used in this review was assessed by the Grades of Recommendation, Assessment, Development and Evaluation (GRADE) [30].

## Results

### Characteristics of included studies

**Fig 1:**
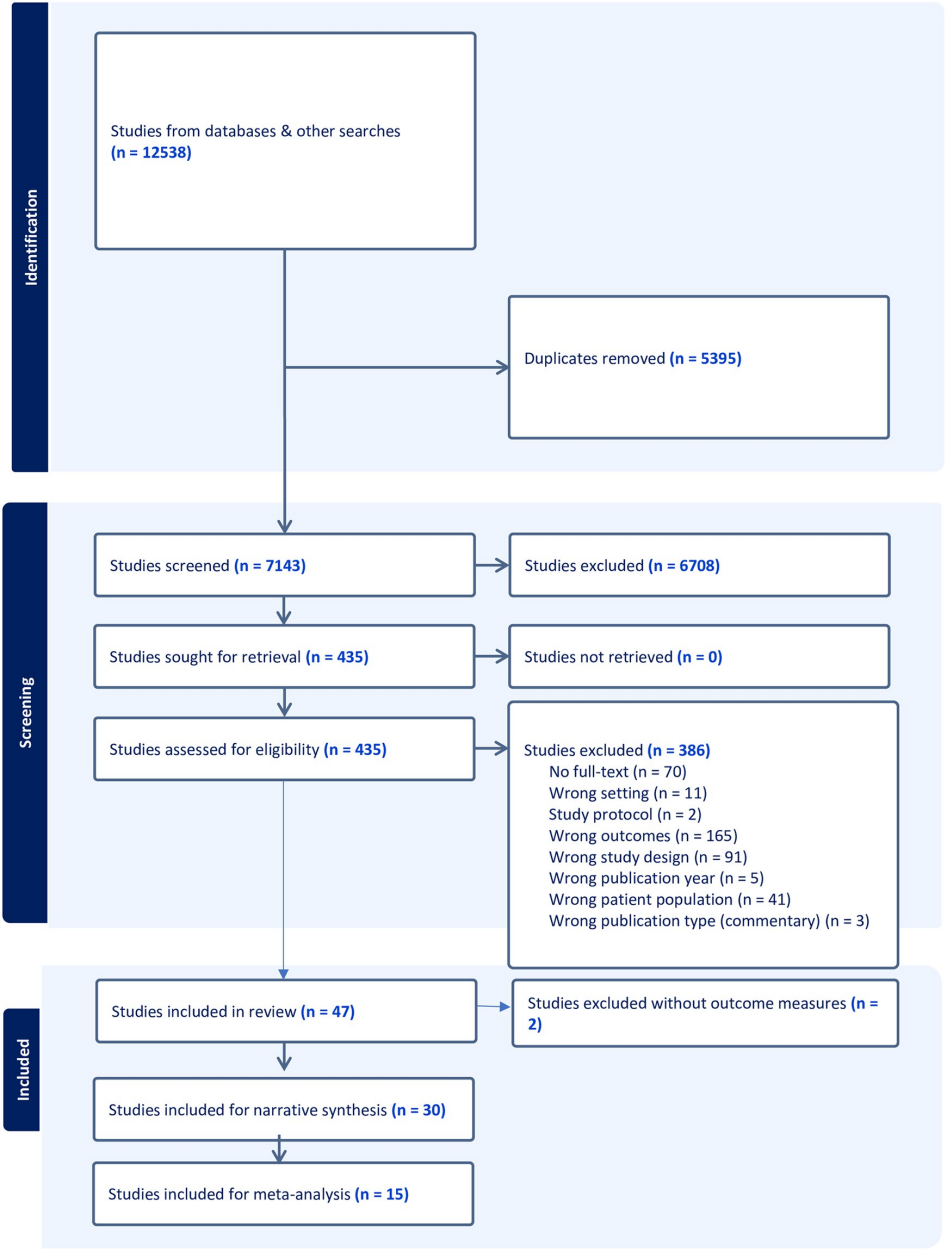
PRISMA flowchart of selection process.

Twelve thousand five hundred and thirty-eight (12538) potential papers were identified for this study. After screening the titles and abstracts, 435 papers were selected for retrieval. When the papers were reviewed, 47 of the 435 papers retrieved for full text screening were found to fully meet our inclusion criteria. The papers were critically appraised using the JBI-MAstARI critical appraisal tool by two independent reviewers and 2 papers were further excluded due to missing outcome measures for different arms of the study. Only 45 papers (30 for narrative synthesis and 15 for meta-analysis) were finally found to be of sufficient quality and were retained (Fig 1).

For the meta-analysis, majority of the studies included were RCTs in design (n = 14), while more than half (16) of the studies included in the narrative synthesis were qualitative in design. Each included study was either conducted only in one SSA country or in two or more SSA countries. Participants in the studies were both adult male and female PLWH aged 15 and above. Details about the studies are summarized in Table 1.

**Table 1:** Characteristics of included studies.

Study (first author, year)	Setting	Study design	Participants/ sample size	Intervention	Comparison	Outcomes	ART barriers	ART facilitators
Ajuna et al., 2021	Two primary health care facilities in southwestern Uganda	Qualitative descriptive study	23 Young adults living with HIV (YALWH). Age: 18–24 years	NA	NA	Barriers and facilitators of ART adherence	Structural barriers to ART adherence (eg, stigma) were the most cited, followed by medication- (eg, pill burden), and patient-related barriers (eg, non-disclosure of HIV status)	Bonding (family, friends, and neighbors), bridging (informal groups), and linking (health professionals) social networks.
Axelsson et al., 2015	Senkatana HIV Clinic in Maseru, Lesotho	Qualitative, semi-structured interview study	28 HIV-positive adults (17 women and 11 men) Age: 18 years and older	NA	NA	Role of timing of ART intake, information patients received from health workers, local adherence patterns, barriers and facilitators of ART adherence	Interruption of daily routines or leaving the house without sufficient medicine	Use of mobile phone alarms, phone clocks and support from family and friends
Azia et al., 2016	Vredenburg regional hospital, Western Cape, South Africa	Descriptive qualitative study	18 non-adhering patients on ART Age: 18–63	NA	NA	Challenges faced by patients on ART	Stigma, disclosure, unemployment, lack of transport, insufficient feeding, disability grants and alternative forms of therapy	NA
Balcha et al., 2011	ART clinics in the Oromia region in Ethiopia	Qualitative study	14 HIV positive patients on ART Age: 26–37 years	NA	NA	Major reasons of patient attrition from treatment at hospital and health center levels.	stigma, financial challenges, and lack of community support, strong belief in holy water and witch doctors among a large segment of the society	disclosure, community support, and decentralization of ART to primary health care units
Becker et al., 2021	Four rural communities in Eswatini	Mixed methods (An exploratory sequential design)	166 rural women living with HIV and receiving ART Age: 20–49 years	NA	NA	Magnitude to which identified barriers impacted ART adherence	household food insecurity, maltreatment by clinic staff, forgetfulness, stress, gossip, mode of transport, age, and lack of community support	NA
Biomndo et al., 2021	Twelve HIV clinics in government health facilities in Kenya	Cross-sectional survey	408 HIV-positive women receiving free ART Age: 18–69 years	NA	NA	Intimate partner violence (IPV) exposure by a current partner, ART adherence rate, and other factors that affect ART adherence	Experiencing physical IPV, sexual IPV, or controlling behavior	Higher education level and having an HIV-positive partner
Bukenya et al., 2019	Kalungu and Wakiso districts in Uganda	Qualitative cross-sectional study	30 individuals on ART Mean age: 41.7 years	NA	NA	How the environmental, social, economic and behavioral experiences of people living with HIV with poor viral suppression could explain their non-adherence to long term ART.	Travel for work or social activities, stigma, receiving little or no continuous ART adherence education, alcohol consumption and use of alternative ‘HIV cure’ medicines, ART side effects, treatment fatigue, belief that long-term ART or God can ‘cure HIV’, and food security	
Buregyeya et al., 2017	Three rural districts (Masaka, Mityana and Luwero) in Uganda	Cross-sectional qualitative study	57 pregnant and breastfeeding women receiving care in six health facilities, who had been on lifelong ART for at least 6 months. Age: 16–42 years	NA	NA	Experiences of HIV infected pregnant and breastfeeding women regarding barriers and facilitators to uptake and adherence to lifelong ART.	Fear to disclose HIV status to partners, drug related factors (side effects and the big size of the tablet), and HIV stigma	Support from male partners and peer family support groups.
Duff et al., 2010	Antenal clinic in a regional hospital in western Uganda	Exploratory, descriptive qualitative study	51 HIV positive Women who had attended antenatal care and received pre-test HIV counselling. Age: 18 years and older	NA	NA	Barriers to accessing and accepting highly active antiretroviral therapy (HAART) by HIV-positive mothers in the Ugandan Kabarole District’s Programme for the Prevention of Mother to Child Transmission-Plus (PMTCT-Plus)	Economic factors (e.g., lack of transportation); social/environmental factors (e.g., stigma towards HIV/AIDS); health care factors (e.g., long waiting times); HIV/HAART knowledge; and HIV disease progression (e.g., physical constraints to attending clinics regularly as an outpatient)	NA
Dzansi et al., 2020	A teaching hospital in Accra, Ghana	A phenomenological qualitative study	26 adult patients receiving ART Age: 18–65 years	NA	NA	Explored adherence behaviour among patients diagnosed with HIV in a teaching hospital in Accra, Ghana	forgetfulnes, secrecy, waiting time, religious beliefs, and sleep.	perception of ART as part of daily routines, benefits of the ART, awareness of regimen, access to food, and transparency.
Essomba et al., 2015	L’hôpital de référence Laquintinie de Douala, Cameroon	Cross-sectional analytic study	521 PLWHIV Age: 15 years and above	NA	NA	Explored factors associated with nonadherence to ART among PLWHIV	Forgetfulness, rupture of medication in hospital, business with other things	NA
Idindil et al., 2012	Tumbi Hospital and Chalinze Health Centre in Pwani Region in eastern Tanzania	Case-control study	316 adult HIV infected people (79 cases and 237 controls matched by age and sex were studied) Age: 18–75 years	NA	NA	Explored factors associated with non-adherence among HIV-infected individuals in Pwani Region	Lack of bus fare, disclosure to confidants only and failure to disclose HIV-test positive status, alcohol use, not satisfied with providers	NA
Kagee et al., 2012	Public hospital in South Africa	Qualitative study	10 patients receiving ART (seven men and three women). Age: 22–37 years	NA	NA	Identified structural barriers that influenced adherence among patients who were enrolled in the national ART programme in South Africa	Structural barriers: time away from work, transport expenses, long waiting times and negative experiences with clinic staff. Pill-taking barriers: food insecurity, stigma and discrimination	NA
Kim et al., 2016	one rural and three urban Malawi Ministry of Health (MOH) facilities in Lilongwe District, Malawi	Qualitative study	65 HIV-infected women who were pregnant or postpartum Age: 16–44	NA	NA	Barriers and facilitators to uptake and adherence to ART	Initiation barriers: concerns about partner support, feeling healthy, needing time to think, ART barriers: ART-related side effects and lack of partner support	Encouragement from community health workers, side effects subsiding, decline in health, change in partner, and fear of future sickness, desire to prevent transmission and improve health
Koole et al., 2016	18 purposively selected health facilities in Tanzania, Uganda, and Zambia	Cross-sectional study	4425 ART patients Age: 18 years or older	NA	NA	Reasons patients miss taking their ART, the pro-portion who miss their ART because of symptoms; and the association between symptoms and incomplete adherence.	Forgetting, ART-related hunger or not having enough food and symptoms (feeling sick or uncomfortable because of the ART), lack of transport, being away from home and asked to stop ART by traditional healer.	NA
Mabunda et al., 2019	Letaba HIV clinic, South Africa	Cross-sectional survey	281 HIV-infected young adults Age: 18–35 years	NA	NA	Factors associated with ART nonadherence	Forgetting, feeling good, fear and running out of treatment	NA
Masa et al., 2017	Two rural communities in eastern Zambia	Cross-sectional study	101 people living with HIV (PLHIV) Age: 18–50 years	NA	NA	Barriers and facilitators of antiretroviral therapy adherence	Having monetary debt, non-farming-related occupation, owning more transportation-related assets, living in community with fewer economic opportunities, place of residence	owning more livestock, place of residence, self-perceived health status (rated as better)
Miller et al., 2010	An NGO clinic in Limpopo Province and a public hospital in Gauteng Province, South Africa	Qualitative study	28 adult patients receiving ART Mean age: 31 years	NA	NA	Assessed reasons for ART default	transport costs, time needed for treatment, and logistical challenges, stigma, ART side effects	NA
Mitiku et al., 2013	Hiwot Fana and Jugal hospitals in Ethiopia	Cross-sectional study	239 PLWHIV Age: >18 years	NA	NA	Explored factors for non-adherence to ART	forgetting, traveling, and being busy doing other things	NA
Moomba and Wyk, 2019	Livingstone General Hospital, Zambia	Explorative, qualitative study	42 patients on ART Age: 18 years and above	NA	NA	Assessed social and economic barriers to ART adherence	Economic factors: poverty, unemployment, and the lack of food Social factors: traditional medicine, religion, lack of family and partner support, and disclosure	NA
Mtetwa et al., 2013	Public HIV clinic in Harare, Zimbabwe	Qualitative study	38 HIV-positive sex workers Age: 18–41 years	NA	NA	explored the reasons for non-attendance and the high rate of attrition	Being demeaned and humiliated by health workers, social stigma, being identified and belittled within the health care environment, competing time commitments and costs of transport and some treatment	NA
Ndirangu et al., 2022	Public health clinics and substance use treatment programmes in Cape Town, South Africa	Qualitative study	69 Women living with HIV Age: 18 and 45 years	NA	NA	Barriers to HIV treatment adherence among women	Intrapersonal-level factors (substance use, financial constraints, food insecurity; community-level factors (anticipated and enacted stigma, community violence) and institutional-level factors (patient–provider relationships, health facility barriers, environmental stigma).	NA
Ngarina et al., 2013	Mitra Plus clinic located within the Muhimbili National Hospital compound, Dar es Salaam, Tanzania	Qualitative study	23 HIV-infected women enrolled in the Mitra Plus PMTCT study with a CD4 cell count of < 200/μL Age: 25–45 years	NA	NA	Reasons for poor adherence to antiretroviral therapy postnatally	Lack of motivation after successfully protecting their child from becoming infected, not feeling ill, feeling hopeless, living in poverty, overwhelming demands of everyday life, having to hide their ART due to the stigma of being HIV-infected	NA
Nsoh et al., 2021	Four hospitals in Yaoundé, Cameroon	Cross-sectional study	271 PLHIV Age: 20 years and above	NA	NA	Assessed predictors of treatment interruption amongst PLHIV and motivating factors to treatment adherence	Transport cost and stigmatization	Belief in the discovery of an eminent HIV cure and the desire to raise offspring
Okoronkwo et al., 2013	Adult retroviral clinic of the Nnamdi Azikiwe University Teaching Hospital, Nnewi, southeast Nigeria.	Cross-sectional descriptive surve	221 adult HIV-infected patients Age: 20–49 years	NA	NA	Explored nonadherence factors in relation to their socioeconomic characteristics	Forgetfulness, busy schedule, side effects of drugs, and stigma	NA
Rasmussen et al., 2013	Simão Mendes HIV Treatment Center in Bissau, Guinea-Bissau	Grounded theory design	20 adult, HIV infected individuals receiving ART at a HIV treatment centre Age: 15–65 years	NA	NA	Assessed barriers and facilitators to patient ART adherence	Limited HIV knowledge, treatment-related costs and competing livelihood needs; poor clinic infrastructure; perceived stigma; and traditional practices	Experienced treatment benefits and complementing social networks.
Schatz et al., 2019	Three Level III health centers and the Medical Research Council/Uganda Virus Research Institute (MRC/UVRI) Health Clinic, Uganda	Qualitative study	40 persons living with HIV Age: 50–96 years	NA	NA	Assessed barriers and facilitators to antiretroviral therapy (ART) access and adherence among older Africans	Transportation costs, food insecurity, and healthcare workers’ knowledge, attitudes, and behaviors	High levels of health literacy, the use of a variety of reminder strategies and viewing ART as life-saving
Tsega et al., 2015	ART clinic of the University of Gondar referral hospital, Ethiopia	Cross-sectional study	351 ART patients Age: > 18 years	NA	NA	ART adherence, associated factors and reasons for nonadherence	forgetfulness (29 [43.3%]), missing appointments (14 [20.9%]), running out of medicine (9 [13.4%]), depression, anger, or hopelessness (4 [6.0%]), side effects of the medicine used (2 [3.0%]), and nonbelief in the ART	NA
Wakibi et al., 2011	HIV/AIDS treatment centre in the Kenyatta National Hospital, Kenya Medical Research Institute (KEMRI) and Riruta Health centre in Nairobi, Kenya	Cross-sectional study	416 HIV+ outpatients Age: 18 years or more	NA	NA	Adherence to ART and identified factors responsible for non-adherence	being busy, forgetting, distance of clinic from home and difficulty with dosing schedule	NA
Weiser et al., 2010	AIDS treatment programs in Mbarara and Kampala, Uganda	Grounded theory design	47 HIV-infected adults Age: 18 years or older	NA	NA	How food insecurity interferes with ARV therapy regimens	Food insecurity: intolerable hunger in the absence of food, Side effects of ARVs were exacerbated in the absence of food, Participants believed they should skip doses if they could not afford the added nutritional burden, competing demands between costs of food and medical expenses, forgot medication doses while working.	NA
Maduka & Tobin-West, 2013	A tertiary health care institution in Nigeria	RCT	104 HIV positive clients who had been HAART experienced for at least three months prior to commencement of the study Age: 20–69 years	Adherence counseling and short message reminders (52 participants received monthly adherence counseling and twice weekly short message reminders for four months + standard care).	Standard care (52 participants received only standard care)	Self-reported adherence and CD4+ cell counts measured pre- and post-intervention (At post-intervention, 76.9% of the intervention group and 55.8% of the control group achieved adherence Also, median CD4+ cell count of the intervention group increased from 193 cells/ml to 575.0 cells/ml against 131.0 cells/ml to 361.5 cells/ml in the control group (P = 0.007).	NA	NA
Graham et al., 2020	A research clinic run by KEMRI in Mtwapa, Kenya	RCT	60 gay, bisexual, and other men who have sex with men (GBMSM) living with HIV Age: 18 years or older	The *Shikamana* intervention (combined modified Next Step Counseling by providers with support from trained peers) to improve adherence (27 (45%) of participants were assigned to the intervention (16 ART-experienced and 11 ART-naïve)	Standard care (33 (55%) of participants were assigned to standard care (17 ART-experienced and 16 ART-naïve)	Feasibility, acceptability, safety, and initial intervention effects–differences in self-reported adherence and virologic suppression. (Feedback on feasibility and acceptability was positive based on exit interviews. After adjustment for baseline viral suppression and confounding, the intervention group had 6-fold increased odds of viral suppression during follow-up).	NA	NA
Fahey et al., 2020	Four health facilities in Shinyanga region, Tanzania	RCT	530 Adults with HIV who had started ART Age: 18 years or older	Cash incentive for monthly clinic attendance (172 in the smaller incentive group, and 174 in the larger incentive group, received monthly incentives for 6 months via mobile health technology	Usual care (184 patients received the usual care	Retention in care with viral suppression (At 6 months, approximately 134 (73%) participants in the control group remained in care and had viral suppression, compared with 143 (83%) in the smaller incentive group (risk difference [RD] 9·8, 95% CI 1·2 to 18·5) and 150 (86%) in the larger incentive group (RD 13·0, 4·5 to 21·5); we identified a positive trend between incentive size and viral suppression (p trend = 0·0032), although the incentive groups did not significantly differ (RD 3·2, −4·6 to 11·0))	NA	NA
Mbuagbaw et al., 2012	Yaoundé Central Hospital (YCH) Accredited Treatment Centre (ATC), Yaounde, Cameroon	Randomized two-arm parallel design trial	200 HIV-positive adults on ART Age: 21 years and above	Motivational mobile phone text messages (SMS) (101 participants received a weekly standardized motivational text message e.g “You are important to your family. Please remember to take your medication. You can call us at this number: +237 xxxx xxxx.”)	Usual care (99 participants received the usual care	At 6 months, overall retention was 81.5%. We found no significant effect on adherence by VAS>95% (risk ratio [RR] 1.06, 95% confidence interval [CI] 0.89, 1.29; p = 0.542; reported missed doses (RR 1.01, 95% CI 0.87, 1.16; p>0.999) or number of pharmacy refills (mean difference [MD] 0.1, 95% CI: 0.23, 0.43; p = 0.617 (SMS did not significantly improve adherence to ART).	NA	NA
Magidson et al., 2021	Khayelitsha community, Western Cape, South Africa	RCT	61 HIV positive people on ART Age: 18–65 years	Khanya, a task-shared, peer-delivered behavioral intervention to improve ART adherence (30 participants received a six-session peer-delivered behavioral intervention, each 60 minutes long, that integrates several evidence-based intervention components–behavioral activation, problem solving, motivational interviewing and mindfulness-based relapse prevention)	Enhanced treatment as usual (ETAU) (31 participants received ETAU–initial screening and referral to t	Ninety-one percent of participants (*n* = 56) were retained at six months. The intervention was highly feasible, acceptable, appropriate and delivered with fidelity (>90% of components delivered as intended by the peer). There was a significant treatment-by-time interaction for ART adherence (estimate = −0.287 [95% CI = −0.507, −0.066]), revealing a 6.4 percentage point increase in ART adherence in *Khanya*, and a 22.3 percentage point decline in ETAU.	NA	NA
Linnemayr et al., 2017	Mildmay clinic, Kampala, Uganda	RCT	155 HIV-infected men and women Age: 19–78	Behavioural incentives (in-kind incentives from a choice of one of three items–a coffee mug, an umbrella or a water bottle)	Standard care	individuals receiving incentives were 23.7 percentage points more likely to achieve 90% antiretroviral adherence compared with the control group [95% confidence interval (CI), 6.7–40.7%]. Specifically, 63.3% (95% CI, 52.9–72.8%) of participants in the pooled intervention groups maintained at least 90% mean adherence during the first 9 months of the intervention, compared with 39.6% (95% CI, 25.8–54.7%) in the control group.	NA	NA
Lester et al., 2010	ART clinics in Kajiado and Nairobi, Kenya	RCT	538 HIV-infected adults initiating antiretroviral therapy (ART) in three clinics in Kenya Age: 18 years and above	Mobile phone short message service (SMS) (273 participants received weekly SMS messages from a clinic nurse and were required to respond within 48 h)	Standard care (265 participants received standard care	Adherence to ART was reported in 168 of 273 patients receiving the SMS intervention compared with 132 of 265 in the control group (relative risk [RR] for non-adherence 0·81, 95% CI 0·69–0·94; p = 0·006). Suppressed viral loads were reported in 156 of 273 patients in the SMS group and 128 of 265 in the control group, (RR for virologic failure 0·84, 95% CI 0·71–0·99; p = 0·04). Patients who received SMS support had significantly improved ART adherence and rates of viral suppression compared with the control individuals	NA	NA
Barnabas et al., 2020	Rural and peri-urban settings in South Africa and Uganda	Randomised trial	1315 People living with HIV Age: 18 years or older	community-based delivery of ART	clinic-based ART delivery	Retention in care and viral suppression (Retention at the month 12 visit was 95% (n = 1253). At 12 months, community-based ART increased viral suppression compared with the clinic group (306 [74%] *vs* 269 [63%], RR 1·18, 95% CI 1·07–1·29; p_superiority_ = 0·0005)	NA	NA
Abiodun et al., 2021	HIV clinics in two states in southwest Nigeria	RCT	209 medication-non-adherent adolescents living with HIV Age: 15–19 years	Interactive and tailored short message reminders to enhance antiretro- viral therapy adherence. 105 participants received daily SMS reminder + Received the standard of care and two clinic visit reminders every four weeks	104 participants received the standard of care and two clinic visit reminders every four weeks. one SMS reminder each for follow-up appointments, 48 hours and 24 hours before the follow-up visit date	ART adherence assessed using the visual analog scale, viral suppression, pill count, and ACTG scores; the feasibility of the intervention by the SMS delivery and response (overall and individual) rates; and acceptability using self-report, willingness to continue receiving the intervention, and desire for its scale-up. (Viral load parameters showed statistically significant differences between control and intervention groups—mean difference for viral load between the two groups was 30,998.42 (95% CI 903.35e61, 093.48) copies/ ml with a p-value of .044).	NA	NA
Orrell et al., 2015	Hannan Crusaid Treatment Centre (HCTC) in Gugulethu, Cape Town, South Africa	RCT	230 ART-naive adults and adolescents Age: ≥15 years	Real-time electronic adherence monitoring device that records dosing time (115 participants received SoC and automated text reminders if dosing >30 minutes late)	standard of care (SoC) (115 participants received 3 pretreatment education sessions)	Median adherence was 82.1% (interquartile range, 56.6%–94.6%) in the intervention arm, compared with 80.4% (interquartile range, 52.8%–93.8%) for SoC [adjusted odds ratio for adherence 1.08; 95% confidence interval (CI): 0.77 to 1.52]. Text message reminders linked to late doses detected by real-time adherence monitoring did not significantly improve adherence or viral suppression.	NA	NA
Peltzer et al., 2012	Ladysmith Hospital in the Uthukela District of KwaZulu-Natal, South Africa	RCT	152 HIV-infected adults on ART Age: 18 years and above	Lay health worker lead structured group intervention (76 participants received three monthly 1 h sessions of medication information combined with problem-solving skills in an experiential/interactive group format)	Standard care (76 participants individually attend monthly one visit to review their health status with their medical practitioner– 20 minutes, standard of care)	There was a significant improvement of ART adherence and CD4 count and a significant reduction of depression scores over time in both intervention and control conditions; however, no significant intervention effect between intervention and control conditions was found.	NA	NA
Pop-Eleches et al., 2011	Chulaimbo Rural Health Center (CRHC) in Nyanza Province, Kenya	RCT	720 Adult patients who had initiated ART Age: > 18 years	Short message service (SMS) reminders (70 participants received short daily reminder messages, 72 received long daily reminders, 73 received short weekly reminders, and 74 received long weekly reminders)	No SMS (139 participants received no SMS)	53% of participants receiving weekly SMS reminders achieved adherence of at least 90% during the 48 weeks of the study, compared with 40% of participants in the control group (*P* = 0.03)	NA	NA
Reid et al., 2017	Independence Surgery, an urban private clinic in Gaborone, Botswana	RCT	108 treatment-experienced adult patients Age: 21 years and above	Cellular phone short message service (SMS) reminders (54 participants received SMS reminders three days prior, one day prior, and the morning of the scheduled monthly pharmacy pickup)	No intervention (54 participants received no SMS)	There were no significant differences in the changes of CD4 count between the intervention and control groups. While mean log HIV VL was lower in the intervention group at the end of six months (5.31 vs. 3.88, *p* = 0.05), the difference in VL from baseline across the groups was not statistically significant (−0.24 vs. 0.09, *p* = 0.14).	NA	NA
Sumari-de Boer et al., 2021	Kilimanjaro Christian Medical Centre and Majengo Health Centre, Tanzania	RCT	249 PLHIV Age: 18–65 years	Digital adherence tools (DATs)–Real-time medication monitoring device + SMS reminder (80 participants received reminder short message service (SMS) texts, followed by a question SMS. 82 participants received a real-time medication monitoring (RTMM) device (Wisepill) with SMS reminders)	Standard care only (81 participants received standard care only)	The average (over 48 weeks) adherence in the SMS, RTMM, and control arms was 89.6%, 90.6%, and 87.9% for pharmacy refill; 95.9%, 95.0%, and 95.2% for self-report in the past week; and 97.5%, 96.6%, and 96.9% for self-report in the past month, respectively (*P* values not statistically significant).	NA	NA
Tirivayi et al., 2012	four Lusaka public-sector ART clinics that distributed food rations (Mtendere, Chawama, Kanyama, and George) and four control clinics that did not distribute rations (Bauleni, Chipata, Matero Reference, and Chilenje), Zambia	Cohort study	400 ART patients receiving food assistance Age: 18 years and above	Food assistance (144 participants received food assistance	No food assistance (147 participants received no food assistance	After 6 months, food assistance recipients (n = 145) had higher ART adherence compared to non-recipients (n = 147, 98.3% versus 88.8%, respectively; p<0.01), but no significant effects were observed for weight or CD4+ lymphocyte count change.	NA	NA

### Quality appraisal

Tables 2 and 3 presents the quality appraisal of studies included in this review. For the included non-randomized control studies, 17 of them had an overall high rate of methodological quality (>90%), 13 had a moderate methodological quality ranging between 80 and 90% and only one had a methodological quality rate of less than 80%. The variation in methodological quality was because some studies did not adequately explain how confounding factors were delt with, did not sufficiently explain participant follow up and the criteria used to objectively assess outcomes in a reliable way (Table 2).

**Table 2:** JBI critical appraisal results for non-randomize control trial studies.

Study	Q1	Q2	Q3	Q4	Q5	Q6	Q7	Q8	Q9	Total (%)
Ajuna, 2021	1	1	0	1	1	N/A	1	1	1	87.5*
Axelsson, 2015	1	1	0	1	1	N/A	1	1	1	87.5*
Azia, 2016	1	1	0	1	1	N/A	1	1	1	87.5*
Balcha, 2011	1	1	0.5	1	1	N/A	1	1	1	93.8*
Becker, 2021	1	1	1	1	1	N/A	1	1	1	100.0*
Biomndo, 2021	1	1	1	1	1	N/A	1	1	1	100.0*
Bukenya, 2019	1	1	0	0.5	1	N/A	1	0.5	1	75.0*
Buregyeya, 2017	1	1	0	1	1	N/A	1	1	1	87.5*
Duff, 2010	1	1	0	1	1	N/A	1	1	1	87.5*
Dzansi, 2020	1	1	0.5	1	1	1	1	1	1	94.4
Essomba, 2015	1	1	1	1	1	N/A	1	1	1	100.0*
Idindil, 2012	1	1	1	1	1	1	1	1	1	100.0
Kagee, 2012	1	1	0	1	1	N/A	1	1	1	87.5*
Kim, 2016	1	1	0	1	1	N/A	1	1	1	87.5*
Koole, 2016	1	1	1	1	1	N/A	1	1	1	100.0*
Mabunda, 2019	1	1	1	1	1	N/A	1	1	1	100.0*
Masa, 2017	1	1	1	1	1	N/A	1	1	1	100.0*
Miller, 2010	1	1	0.5	1	1	1	1	1	1	94.4
Mitiku, 2013	1	1	1	1	1	N/A	1	1	1	100.0*
Moomba, 2019	1	1	0	1	1	N/A	1	1	1	87.5*
Mtetwa, 2013	1	1	0	1	1	N/A	1	1	1	87.5*
Ndirangu, 2022	1	1	1	1	1	1	1	1	1	100.0
Ngarina, 2013	1	1	0	1	1	N/A	1	1	1	87.5*
Nsoh, 2021	1	1	1	1	1	1	1	1	1	100.0
Okoronkwo, 2013	1	1	1	1	1	N/A	1	1	1	100.0*
Rasmussen, 2013	1	1	0	1	1	N/A	1	1	1	87.5*
Schatz, 2019	1	1	0	1	1	N/A	1	1	1	87.5*
Tsega, 2015	1	1	1	1	1	N/A	1	1	1	100.0*
Wakibi, 2011	1	1	1	1	1	0.5	1	1	1	94.4
Weiser, 2010	1	1	0	1	1	N/A	1	1	1	87.5*
Tirivayi, 2012	1	1	1	1	1	1	1	1	1	100.0

1 = Yes, 0 = No, 0.5 = Unclear, N/A = not applicable

* Score is out of 8 due to an inapplicable item

**Table 3:** JBI critical appraisal results for randomized controlled trial studies.

Study	Q1	Q2	Q3	Q4	Q5	Q6	Q7	Q8	Q9	Q10	Total (%)
Maduka, 2013	1	1	1	1	1	1	1	1	1	1	100.0
Graham, 2020	1	1	1	0.5	1	1	1	1	1	1	95.0
Fahey, 2020	1	1	0.5	N/A	0.5	1	1	1	1	1	88.9*
Mbuagbaw, 2012	1	1	1	1	1	1	1	1	1	1	100.0
Magidson, 2021	1	1	1	1	0.5	1	1	1	1	1	95.0
Linnemayr, 2017	1	1	0.5	0.5	0.5	1	1	1	1	1	85.0
Lester, 2010	1	1	1	0	1	1	1	1	1	1	90.0
Barnabas, 2020	1	1	0	0	0	1	1	1	1	1	70.0
Abiodun, 2021	1	1	1	0	1	1	1	1	1	1	90.0
Orrell, 2015	1	1	1	0	0.5	1	1	1	1	1	85.0
Peltzer, 2012	1	1	0.5	0	0.5	1	1	1	1	1	80.0
Pop-Eleches, 2011	1	1	0.5	0	0.5	1	1	1	1	1	80.0
Reid, 2017	1	1	1	N/A	0	1	1	1	1	1	88.9*
Sumari-de Boer, 2021	1	1	0	0	0	1	1	1	1	1	70.0

1 = Yes, 0 = No, 0.5 = Unclear, N/A = not applicable

* Score is out of 9 due to an inapplicable item

For the included randomized control trials, 6 had a high methodological quality rate (>90%), 6 had a rate between 80–90% and 2 had a methodological quality rate of less than 80%. The variations in methodological quality among trials was because some trials did not conceal the allocation of treatment from allocators, outcomes of participants who withdrew from the trials were not described nor included in the analysis and those assessing outcomes were not blind to treatment allocation (Table 3).

### Common barriers and facilitators to ART adherence in SSA

Thirty studies [18, 31–59] reported barriers to ART adherence and 11 of the studies [31–34, 37, 39, 43, 46, 53, 55, 56] also reported facilitators to ART adherence. Both the barriers and facilitators can be grouped under the same seven broad categories–patient, health system, medication, stigma, poor mental health, socio-economic and socio-cultural-related factors (Table 4). Specific details of the studies with the barriers and facilitators reported can be found on Table 1.

**Table 4:** Common barriers and facilitators to ART adherence in SSA.

Category	Barriers	Facilitators
Patient-related factors	Travel for work or social activities Business with other things Forgetfulness Perceived wellness or feeling healthy HIV disease progression Non status disclosure Limited HIV/ART knowledge use of alcohol or other drugs Age Stress Leaving house without sufficient drugs	Use of reminders Having routines Disclosing status HIV/ART education
Health system-related factors	Poor relationship with care providers Poor clinic infrastructure Health workers knowledge, attitudes and behaior Long waiting times Poor service delivery No continuous ART adherence education Rupture of drugs	Caregiver support Decentralization of ART to primary care units Improved relationship with care providers
Medication-related factors	Side effects Pill burden/ dosing Big size of tablet Treatment fatigue ART-related hunger	Awareness of regimen Benefit of ART
Stigma-related factors	Gossips Fear of status disclosure	Accept status View ART as lifesaving Improve knowledge and understanding of HIV/ART
Poor mental health-related factors	Depression Anger or hopelessness Feeling overwhelmed with demands of daily life Sleep disorders	Motivation to be healthy for self and others Counseling Belief in eminent HIV cure discovery Desire to raise offsprings
Socio-economic-related factors	Unemployment Poverty Lack of partner/family support No disability grants Food insecurity	Disability grants Financial support
Socio-cultural-related factors	Use of alternative medicines Intimate partner violence Community violence Religious beliefs/practices Belief in witch doctors Lack of community support	Partner/family support Social support Community support Encouragement from community health workers

### Common ART adherence interventions and their outcome in SSA

Common interventions used in improving ART adherence in SSA countries include counselling [60, 61], incentives [62, 63], mobile phone short message service (SMS) [60, 64–69], peer delivered behavioural intervention [70], community ART delivery intervention [71], electronic adherence monitoring device [69, 72], lay health worker lead group intervention [73] and food assistance [74]. The counselling intervention (either used alone or in combination with another intervention such as SMS), peer delivered intervention, food assistance, community ART delivery as well as lay health worker lead group interventions have a significant effect in improving patients’ adherence to ART. However, incentives (either financial or in kind), SMS and electronic adherence monitoring device interventions have varying statistically significant or non-significant effects in different settings (Table 5).

**Table 5:** ART adherence interventions and their outcomes in SSA.

Study	Intervention	Control	Outcome of intervention
Maduka, 2013	Adherence counseling and short message reminders	Standard care	Significant effect on ART adherence and CD4 cell count
Graham, 2020	The *Shikamana* intervention (combined modified Next Step Counseling by providers with support from trained peers) to improve adherence	Standard care	Significant effect on viral suppression
Fahey, 2020	Cash incentive for monthly clinic attendance	Usual care	Significant effect on viral suppression
Mbuagbaw, 2012	Motivational mobile phone text messages (SMS)	Usual care	No significant effect on adherence
Magidson, 2021	Khanya—a task-shared, peer-delivered behavioral intervention to improve ART adherence	Enhanced treatment as usual (ETAU)	Significant treatment-by-time interaction for ART adherence
Linnemayr, 2017	Behavioural incentives (in-kind incentives from a choice of one of three items–a coffee mug, an umbrella or a water bottle)	Standard care	no significant effect on Adherence.
Lester, 2010	Mobile phone short message service (SMS)	Standard care	Significant effect on ART adherence and viral suppression
Barnabas, 2020	Community-based delivery of ART	clinic-based ART delivery	Significant effect on viral suppression
Abiodun, 2021	Interactive and tailored short message reminders to enhance antiretro-viral therapy adherence.	Standard care & two clinic visit reminders every four weeks.	Significant effect on viral load suppression
Orrell, 2015	Real-time electronic adherence monitoring device that records dosing time	standard of care (SoC)	No significant improvement in adherence or viral suppression.
Peltzer, 2012	Lay health worker lead structured group intervention	Standard care	Significant improvement of ART adherence and CD4 count
Pop-Eleches, 2011	Short message service (SMS) reminders	No SMS	Significant effect on ART adherence
Reid, 2017	Cellular phone short message service (SMS) reminders	No intervention	No significant differences in the changes of CD4 and viral load
Sumari-de Boer, 2021	Digital adherence tools (DATs)—Real-time medication monitoring device + SMS reminder	Standard care only	No statistically significant difference in ART adherence
Tirivayi, 2012	Food assistance	No food assistance	Significant effect on ART adherence, but no significant effects were observed for weight or CD4+ lymphocyte count

### Effect of interventions on ART adherence—meta-analysis

**Fig 2:**
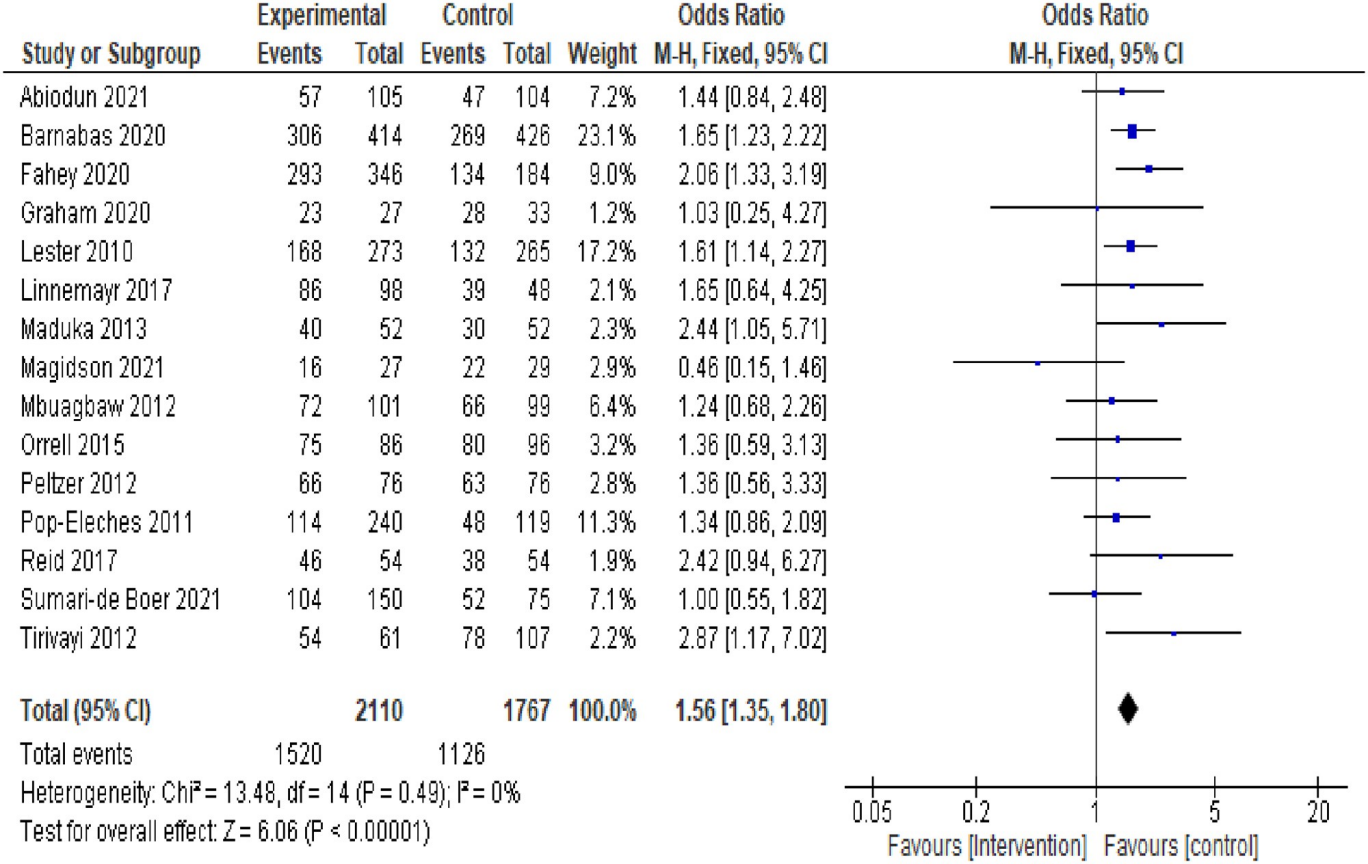
Forest plot of ART adherence comparing adherence interventions versus control.

Fourteen RCTs and 1 cohort study reported ART adherence as an outcome. All 15 included studies were pooled in the meta-analysis. The total number of participants in the pooled studies were 3877 PLWH who were on ART. Of these 3877 participants, 2110 received an ART adherence intervention and 1520 of them were ART adherent after the intervention. Overall, the results of the pooled analysis from both the RCTs and cohort study included showed a statistically significant difference in ART adherence rates between the intervention and controlled groups (pooled OR = 1.56, 95%CI:1.35–1.80, p = <0.01), with evidence of low none statistically significant heterogeneity between studies (*I*^*2*^ = 0%, p = 0.49) (Fig 2).

**Fig 3:**
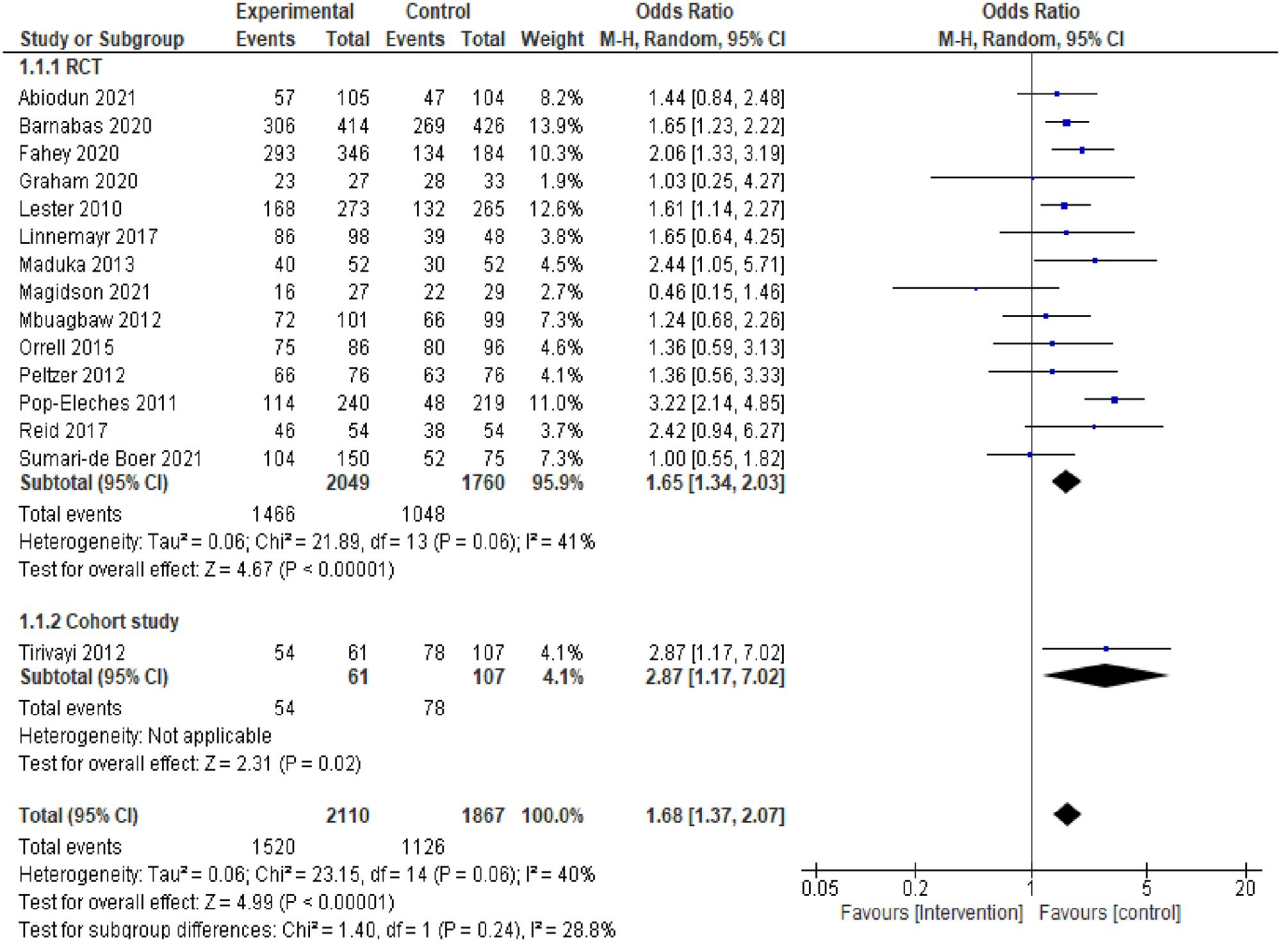
Forest plot of ART adherence comparing adherence interventions versus control in subgroup RCTs and cohort study analysis.

In the subgroup analysis of RCTs and cohort study, the individual and pooled ORs for ART adherence are shown in Fig 3. The results of the pooled analysis from the RCTs showed a statistically significant difference in ART adherence between the intervention and control groups (pooled OR = 1.65, 95%CI:1.34–2.03, p = <0.01), with evidence of moderate none statistically significant heterogeneity between studies (*I*^*2*^ = 41%, p = 0.06). Similarly, the result of the analysis from the cohort study showed a statistically significant difference in ART adherence between the intervention and control groups (OR = 2.87, 95%CI: 1.17–7.02; p = 0.02).

**Fig 4:**
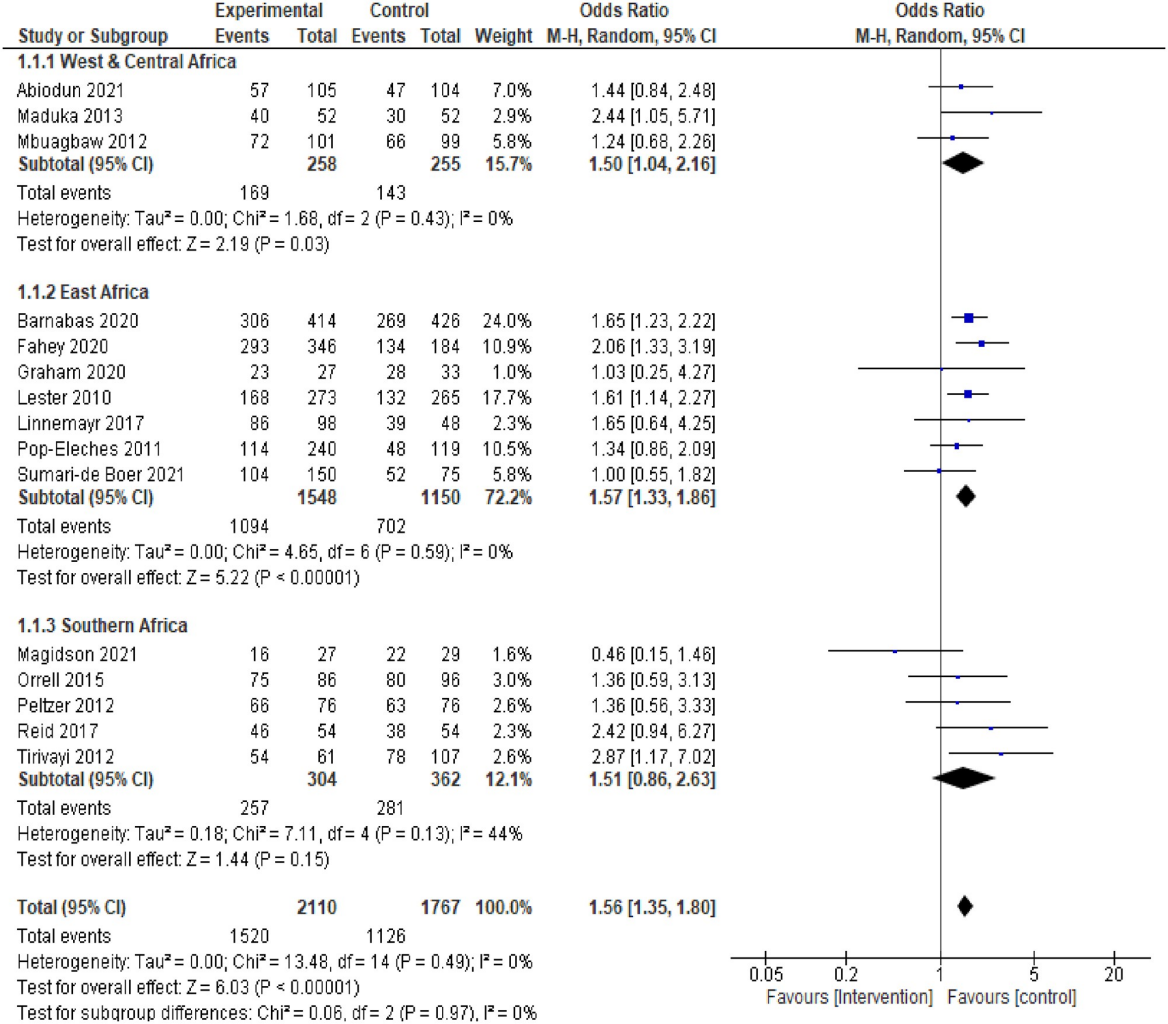
Forest plot of ART adherence comparing adherence interventions versus control in subgroup analysis by region.

Furthermore, in the subgroup analysis of studies by region (West and Central African region, East African region, and Southern African region), the results of the pooled analysis from studies in the West and Central African as well as those from the East African region showed a statistically significant difference in ART adherence between intervention and control groups but with no statistically significant heterogeneity between studies. A moderate none statistically significant heterogeneity (*I*^*2*^ = 44%, p = 0.13) was only observed in the Southern African region studies which also showed a none statistically significant difference in ART adherence between intervention and control groups ((pooled OR = 1.51, 95%CI:0.86–2.63, p = 0.15), (Fig 4).

### Confidence in the evidence

The assessment of the quality of the evidence used in this review shows that there is high certainty of evidence in intervention effect on the domain of patients’ adherence to ART. The certainty of evidence on the outcome domains of retention in care and CD4 cell counts is low. Lastly, certainty of evidence on the viral load outcome domain is very low (Table 6).

**Table 6:** GRADE certainty of evidence ratings.

Outcome domain/ studies	Sub-outcomes	Methodology	Inconsistency	Indirectness	Imprecision	Publication bias	Level of certainty of evidence
Adherence to ART [Abiodun, Maduka, Mbuagbaw, Reid, Lester, Linnemayr, Magidson, Pop-Eleches, Tirivayi, Peltzer, Sumari-de Boer]	a. self-reported adherence b. pharmacy pickups c. pharmacy refill d. pill count or tablet return	No serious concerns	No serious concerns	No serious concerns	No serious concerns	Minor concerns (some studies did not disaggregate sub-outcomes)	HIGH CERTAINTY—⊕⊕⊕⊕ (minor concerns about publication bias)
Retention in care [Barnabas, Pop-Eleches, Fahey, Magidson, Mbuagbaw, Graham]	a. proportion of patients retained after intervention	No serious concerns	Serious concerns (some heterogeneity in outcomes of interest)	No serious concerns	Minor concerns (lack of confidence intervals in some results)	No serious concerns	LOW CERTAINTY—⊕⊕OO (serious concerns with inconsistency and minor concerns with imprecision)
CD4 cell counts [Graham, Maduka, Peltzer, Reid, Tirivayi]	a. increase in CD4 cell count	No serious concerns	Minor concerns (only five studies)	No serious concerns	Minor concerns (some studies did not report actual CD4 cell counts)	No serious concerns	LOW CERTAINTY—⊕⊕OO (minor concerns with inconsistency and imprecision)
Viral load [Abiodun, Lester, Reid, Orrell, Fahey, Graham, Barnabas]	a. undetected viral load (< 40 copies /ml)	No serious concerns	Very serious (some heterogeneity in outcomes of interest)	Minor concerns (some studies did not report direct outcomes)	Minor concerns (mixed findings in terms of viral load copies/ml)	Minor concerns (some studies did not disaggregate sub-outcomes)	VERY LOW—⊕OOO (serious concerns with inconsistency, indirectness, imprecision, and publication bias)

⊕⊕⊕⊕—high certainty, ⊕⊕⊕O—moderate certainty, ⊕⊕OO—low certainty, ⊕OOO—very low certainty.

## Discussion

ART regimens are life-long requirement of strict compliance by patients and needs to be taken as prescribed to achieve treatment success and prevent drug resistance [7, 8, 75]. As clinical and immunological improvement as well as viral suppression are only expected when individuals adhere to ART [5, 7–9], the efficacy and durability of ART drug regimens require near perfect adherence rates as high as 95% or more [76–78].

This systematic review and meta-analysis synthesized existing evidence on ART adherence barriers, facilitators and strategies or interventions for improving patients’ adherence to ART in SSA countries. We document that 30 studies included in this review reported barriers to ART adherence, 11 of them also reported facilitators to ART adherence and 15 studies reported interventions improving ART adherence in SSA. The common barriers reported are grouped under seven major factors: patient-related (age, use of alcohol or other drugs, forgetfulness, business with other things, non-status disclosure, limited HIV knowledge, leaving house without drugs, perceived wellness or feeling healthy), health system-related (poor clinic infrastructure, health workers knowledge and attitudes, long waiting times, poor service delivery, rupture of drugs), medication-related (side effects, pill burden/dosing, treatment fatigue), stigma (gossips, fear of status disclosure), poor mental health (depression, anger and hopelessness, sleep disorders, feeling overwhelmed with life demands), socioeconomic (unemployment, poverty, food insecurity, no disability grants, lack of partner/family support) and socio-cultural-related (use of alternative medicines, intimate partner violence, religious beliefs/practices, belief in witch doctors, lack of community support) factors. Similarly, the common facilitators to ART adherence are also group under the same seven major factors as barriers and they include: patient-related (use of reminders, having routines, disclosing status and HIV/ART education), health system-related (caregiver support, decentralization of ART care units, improved relationship with care providers), medication-related (awareness of regimen, benefits of ART), stigma (accept status, view ART as life-saving, improve knowledge and understanding of HIV/ART), poor mental health (motivation to be healthy for self and others, counselling, belief in eminent HIV cure discovery, desire to raise offspring), socioeconomic (disability grants, financial support) and socio-cultural-related (partner/family support, social support, community support, encouragement from community health workers). The interventions improving ART adherence include counselling, incentives, mobile phone short message service (SMS), peer delivered behavioural intervention, community ART delivery intervention, electronic adherence service monitoring device, lay health worker lead group intervention and food assistance. These interventions are either effective individually or when combined and the results of our meta-analysis revealed an improvement in ART adherence in favour of ART interventions.

There have been several reviews in the realm of ART adherence, each with its distinct scope and limitations. Some are somewhat dated, others might have methodological constraints, and a significant portion focuses exclusively on English-language publications. Specifically: a 2008 review centred on barriers to accessing antiretroviral treatment in developing nations, emphasizing studies from 1996 to 2007 and exclusively sourcing from PubMed, FamMed and Cochrane databases [79]. A 2012 examination reviewed ART adherence trends in Cameroon, covering studies between January 1999 and May 2012 [80]. Another review from 2003 to February 2019 addressed the efficacy of treatment supporter interventions for ART adherence in SSA, but it considered only English-language studies [81]. A further review regarding strategies to bolster adherence in sub-Saharan Africa exclusively considered articles from PubMed, Medline and Google Scholar databases [82].

Our study fills a unique niche in this spectrum: it provides an all-encompassing view of barriers, facilitators, and interventions to enhance ART adherence in SSA, embracing both English and French publications. This makes our review distinct as no similar comprehensive study has been identified.

Delving into our findings, we observed that the reported barriers align with those from other reviews, specifically those relating to patient factors, medication, stigma and health services [25]. Nonetheless, our review offers a broader scope, encompassing barriers that have been somewhat overlooked by others, like transport [83] and food insecurity issues [84]. Other reviews focused exclusively on population and health system level barriers [79] and depression and alcohol use related barriers [85]. Furthermore, our findings regarding facilitators mirror those from other reviews, noting common themes like social support, reminders, status disclosure and the importance of establishing robust patient-provider relationships [86].

Regarding interventions, our review highlights a variety of strategies, some potent as standalone measures, and others more effective in tandem with complementary interventions. This is consistent with another review asserting the necessity of multiple interventions due to the diverse barriers faced by ART patients [25]. While our review doesn’t single out the most impactful intervention, our meta-analysis underscores an overarching improvement in adherence when ART interventions are implemented. This is in line with other analyses which have demonstrated increased adherence with the introduction of interventions [87]. It is also noteworthy that some specific groups, like pregnant and lactating women, show heightened adherence when given targeted interventions [88]. Community-based ART delivery, as observed in other reviews, also emerges as a potent strategy, enhancing patient retention, accessibility to HIV services, and overall treatment engagement [89–91].

### Strengths and limitations

This review has the following strengths–eligible studies were identified through a comprehensive search on several databases and sources, it included recent articles published in SSA countries from 2010 onward, studies published in either English and or French were included, and two persons independently evaluated each study for inclusion and data extraction. Furthermore, this review alone has synthesized recent evidence on common adherence barriers and facilitators, and common interventions that have been shown to improve ART adherence across SSA in one single review than any previous review has done.

However, our review is limited by the fact that we included some cohort studies which may bias the overall estimate effect due to unmeasured confounding not adjusted for in multivariable analysis. Also, included studies for our meta-analysis did not measure the outcome (adherence) in the same way nor using the same tool; this might impact the results of our pooled analysis. Lastly, we did not include unpublished studies and might have missed some eligible articles.

## Conclusion

The barriers and facilitators to ART adherence in SSA countries can be categorized under seven primary factors: patient-related, health system-related, medication-related, stigma, poor mental health, socioeconomic and socio-cultural-related factors. Common interventions that enhance ART adherence encompass counselling, incentives, mobile phone short message services (SMS), peer delivered behavioural intervention, community ART delivery intervention, electronic adherence service monitoring device, lay health worker lead group intervention and food assistance. These strategies prove effective either as standalone approaches or in conjunction with other methods. To harness the full potential of ART and mitigate the HIV burden in SSA countries, stakeholders engaged in HIV prevention and treatment must recognize and integrate these barriers, facilitators, and adherence enhancing interventions when formulating policies or crafting treatment strategies. Ensuring sustained optimal outcomes from ART may necessitate further research, particularly focusing on specific underrepresented demographics such as HIV-infected children, adolescents, and pregnant women in SSA. This research will aim to uncover the most appropriate barriers, facilitators and interventions tailored to each group’s unique needs.

## Supporting information

S1 File PRISMA 2020 checklist. (DOCX)

S2 File Medline (Ovid) search strategies and results. (DOCX)

S3 File Joanna Briggs institute for meta-analysis of statistics assessment and review instruments. (DOCX)

S4 File Data extraction form for quantitative research. (DOCX)

## Abbreviations

AIDS: Acquired Immune deficiency syndrome
ART: Antiretroviral Therapy
CI: Confidence interval
GRADE: Grading of Recommendations Assessment, Development and Evaluation
HIV: Human immunodeficiency virus
JBI: Joanna Briggs Institute
JBI-MASTARI: Joanna Briggs Institute Meta-Analysis of Statistics Assessment and Review instrument
LMIC: Low and Middle-Income Countries
OR: Odds Ratio
PLWH: People living with HIV
PLWHA: People living with HIV/AIDS
PRISMA-P: Preferred Reporting Items for Systematic Reviews and Meta-Analyses Protocols
PROSPERO: International Prospective Register of Systematic Reviews
SSA: Sub-Saharan Africa
