# Supplementary material for: Barriers and facilitators for interventions to improve ART adherence in Sub-Saharan African countries: A systematic review and meta-analysis
Source: PLoS One. 2023 Nov 30;18(11):e0295046. doi: 10.1371/journal.pone.0295046 (PMC10688728; doi:10.1371/journal.pone.0295046)
Supplement: S3 File — (DOCX) [file pone.0295046.s003.docx]

**S2_File: Joanna Briggs Institute for Meta-Analysis of Statistics Assessment and Review Instruments**

1. JBI critical appraisal checklist for randomized control trials

Reviewer……………………………………….. Date……………………………………..

Author………………………………………….. Year……………………………………..

|  |  | Yes | No | Unclear | Not applicable |
| --- | --- | --- | --- | --- | --- |
| 1 | Was the assignment and treatment groups truly random? |  |  |  |  |
| 2 | Were participants blind to treatment allocation? |  |  |  |  |
| 3 | Was the allocation to treatment groups concealed from the allocator? |  |  |  |  |
| 4 | Were the outcomes of people who withdrew described and included in the analysis? |  |  |  |  |
| 5 | Were those assessing outcomes blind to the treatment allocation? |  |  |  |  |
| 6 | Were the control and treatment groups comparable at entry? |  |  |  |  |
| 7 | Were groups treated identically other than for the named interventions? |  |  |  |  |
| 8 | Were outcomes measured in the same way for all groups? |  |  |  |  |
| 9 | Were outcomes measured in a reliable way? |  |  |  |  |
| 10 | Was appropriate statistical analysis used? |  |  |  |  |

Overall appraisal: include……… exclude………. Seek further info……….

Comments (include reasons for exclusion)……………………………………………………..

1. JBI critical appraisal checklist for non-randomized control trials

Reviewer……………………………………….. Date……………………………………..

Author………………………………………….. Year……………………………………..

| 1 | Was study based on random or pseudo sample? |  |  |  |  |
| --- | --- | --- | --- | --- | --- |
| 2 | Were the criteria for inclusion in the sample clearly defined? |  |  |  |  |
| 3 | Were confounding factors identified and strategies to deal with them stated? |  |  |  |  |
| 4 | Were outcomes assessed using objective criteria? |  |  |  |  |
| 5 | If comparisons are being made, was there sufficient description of the groups? |  |  |  |  |
| 6 | Was follow up carried out over a sufficient time period? |  |  |  |  |
| 7 | Were the outcomes of people who withdrew described and included in the analysis? |  |  |  |  |
| 8 | Were outcomes measured in a reliable way? |  |  |  |  |
| 9 | Was appropriate statistical analysis used? |  |  |  |  |

Overall appraisal: include……… exclude………. Seek further info……….

Comments (include reasons for exclusion)……………………………………………………..
